# Supplementary material for: Defect free strain relaxation of microcrystals on mesoporous patterned silicon
Source: Nat Commun. 2022 Nov 4;13:6624. doi: 10.1038/s41467-022-34288-4 (PMC9636155; doi:10.1038/s41467-022-34288-4)
Supplement: Supplementary file 1 — Supplementary Information [file 41467_2022_34288_MOESM1_ESM.pdf]

Supplementary information for

Defect free strain relaxation of microcrystals on mesoporous patterned silicon

Heintz et al.

Correspondence to: *alexandre.heintz@usherbrooke.ca*; *abderraouf.boucherif@usherbrooke.ca*

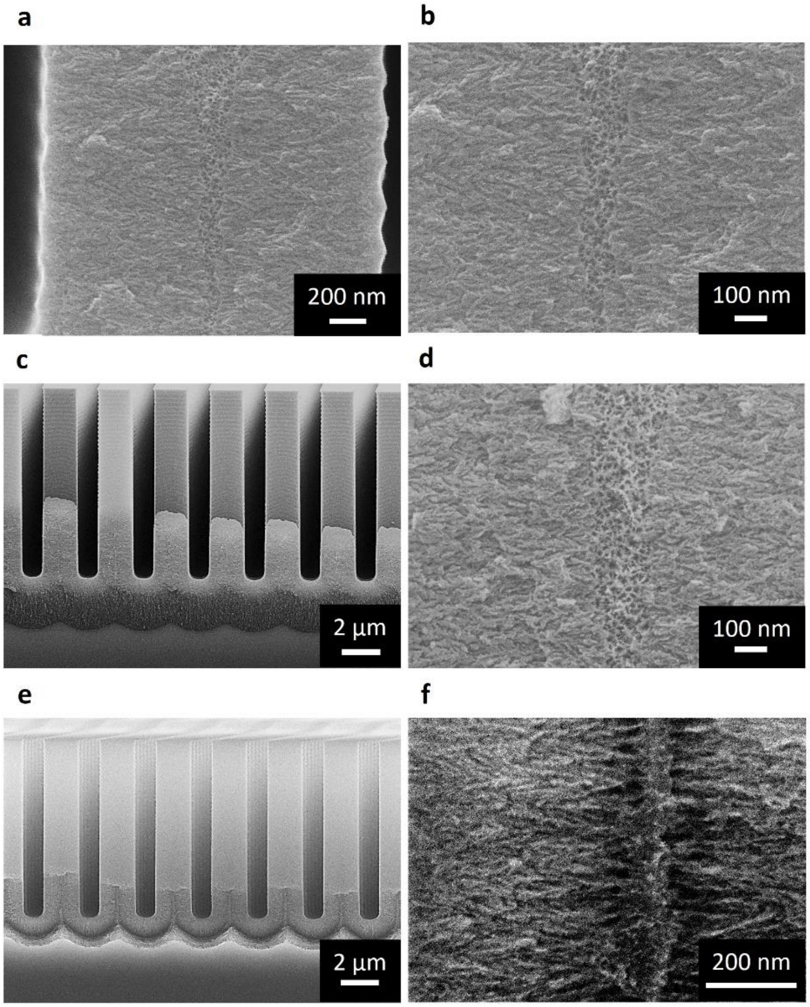

Fig. S1. Synthesis of various mesoporous silicon. Cross section SEM images porous silicon pillars. (a) PSiP(40%), (c) PSiP(50%), (e) PSiP(70%). (b,d,f) High-magnification SEM images of the mesoporous pillar structure. To understand the effect of porosity on lattice strain accommodation, various porosity was studied from 40 to 70 % of porosity to reach suitable Young's Modulus. The etching parameters such as current density, time of porosification and electrolyte concentration were tuned to reach the desired porosity. Due to the patterning, the electrochemical etching occurred in 3 dimensions, the growth of porous start from all free lateral surfaces exposed by the pillar.

Table S1. *Porosification parameters.*

Current density (mA/Cm²)	Electrolyte HF:Eth (Volume :Volume)	Mode	Porosity	Time (s)
50	1:1	Pulse 1s/1s	40-45 %	60
120	1:1	Pulse 1s/1s	50-55%	35
50	1:3	direct	70-75%	150

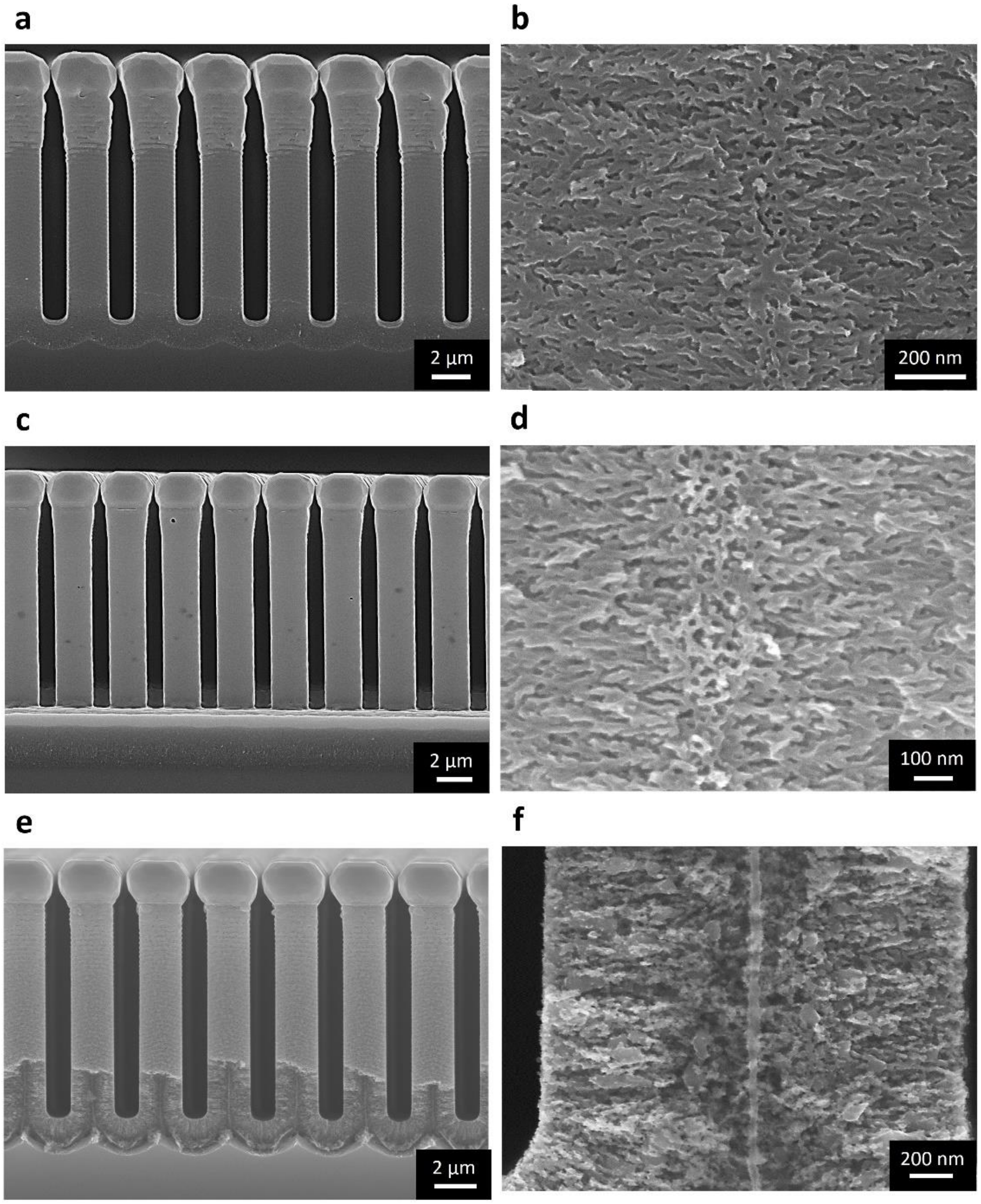

Fig. S2. Ge growth on various porous silicon pillars. Cross section SEM images of Ge microcrystals grown on porous silicon pillars (**a**,**c**,**e**). Cross section SEM images of reorganized porous silicon pillars after Ge growth. (**b**) PSiP(40%), (**d**) PSiP(50%), (**f**) PSiP(70%). The porous Si structure undergoes morphological transformation to reduce its total surface energy, due to the thermal budget provided during Ge epitaxial growth following the Ostwald ripening process. Accordingly, during thermal annealing the Si atoms on the surface of the pores move to energetically favorable positions, leaving Si-free volume into the center of the pillars.

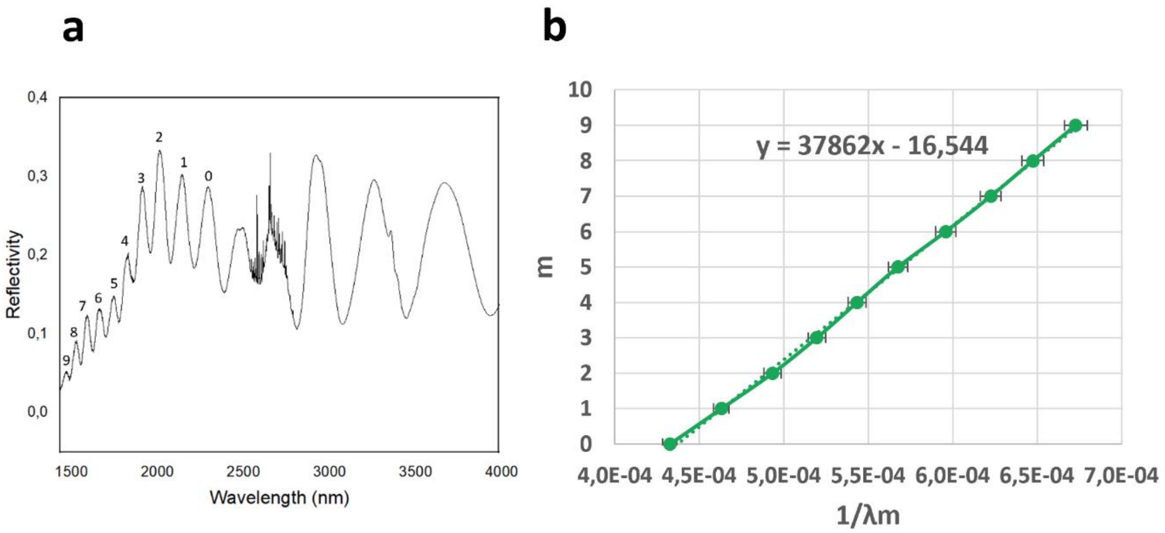

Fig. S3. Fabry-Pérot interference spectrum. Reflectance spectrum of Porous Silicon Pillars with high porosity (**a**), order of the fringes as a function of 1/λm (**b**). The order of the fringe is enumerated from 0 to 9, following the Equation (2) the effective optical thickness can be determine: $2n_{Porous}L$ = 37862. Thus, considering the thickness L= 12 µm of the porous structure, we can calculate the effective refractive index of the porous silicon pillar n_{Porous} = 1.54
Finally, according to Equation (3), the porosity obtained is P = 70.6 %. The result obtained using this method remain close to the porosity determine using ImageJ (70-75%).

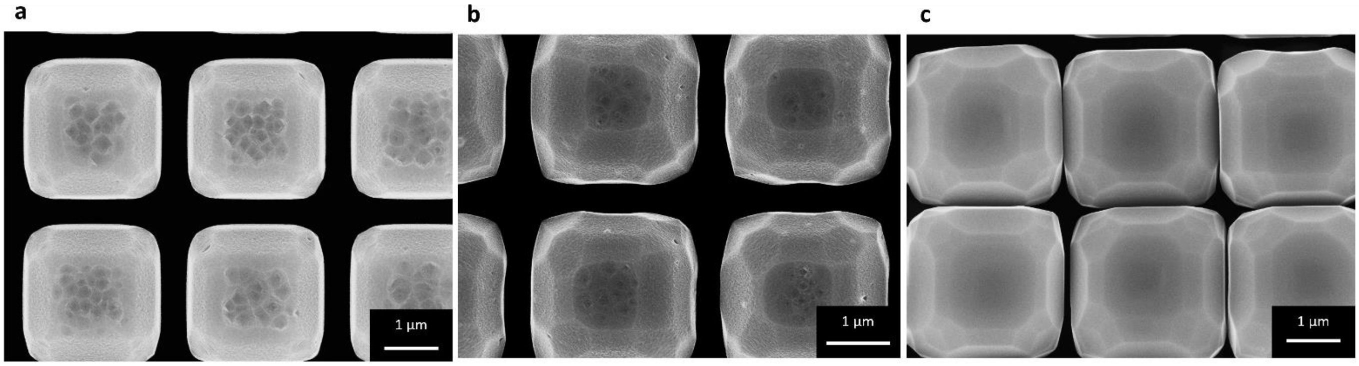

Fig. S4. Etch-Pit method of Ge microcrystal on various substrate. TOP view SEM images of Ge/SiP(**a**), Ge/PSiP(50%)(**b**),Ge/PSiP(70%)(**c**), after Etch-Pit method.

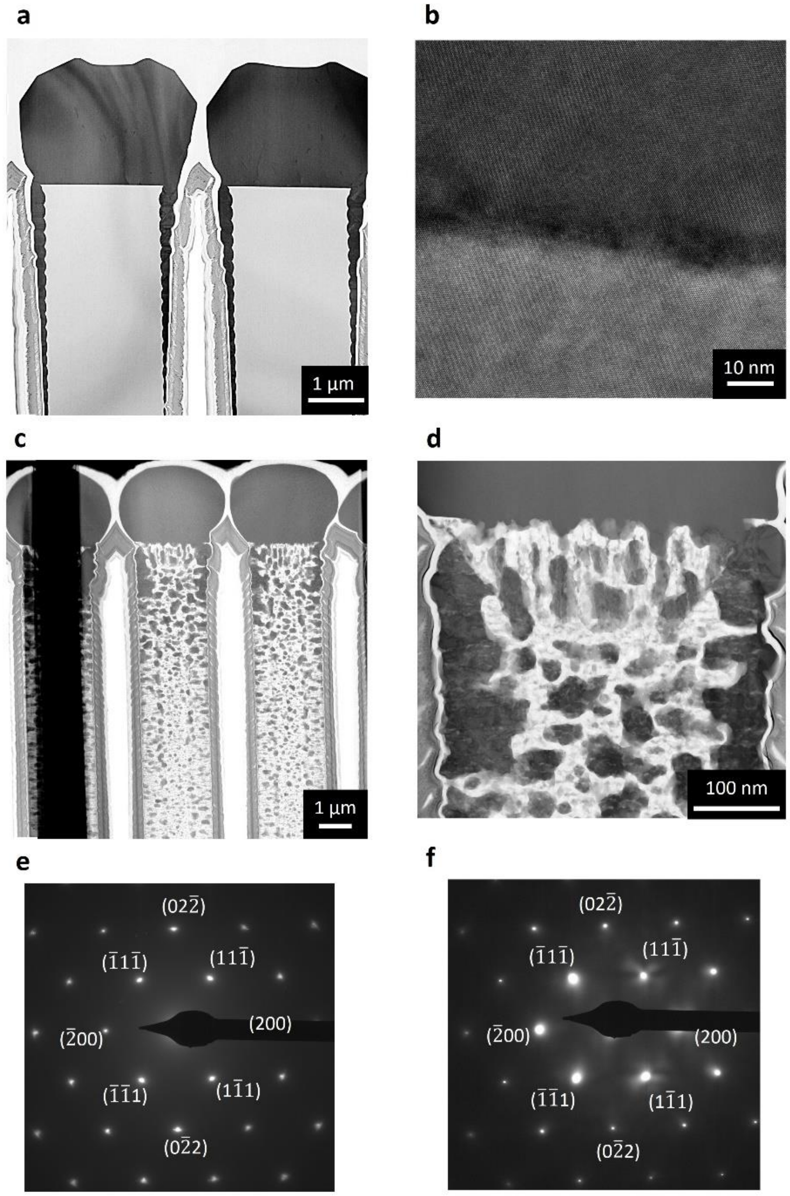

Fig. S5. TEM characterization of the heterostructures. Low magnification BF-TEM images of the Ge/SiP reference sample (**a**), high resolution TEM image of the Ge/SiP interface with misfit dislocation network (**b**). Low magnification BF-TEM image of several Porous silicon Pillars (PSiP) substrates with a defect-free Ge crystals (**c**), the porous structure (**d**). Selected area electron diffraction (SAED) pattern of the relaxed and monocrystalline Ge crystal on the porous structure (**e**), and the SAED pattern of the porous silicon (**f**). TEM observations were performed on several pillars for each Ge grown on SiP and PSiP substrates. The defects appeared in the reference sample with the dislocation misfit network, while Ge microcrystals grown on PSiP substrate remain defect-free. SAED patterns confirm the monocrystalline and relaxed Ge microcrystal obtained on PSiP. Moreover, the deformation of the dots in the SAED pattern related to the substrate shows the lattice accommodation of the porous template and SiGe compound.

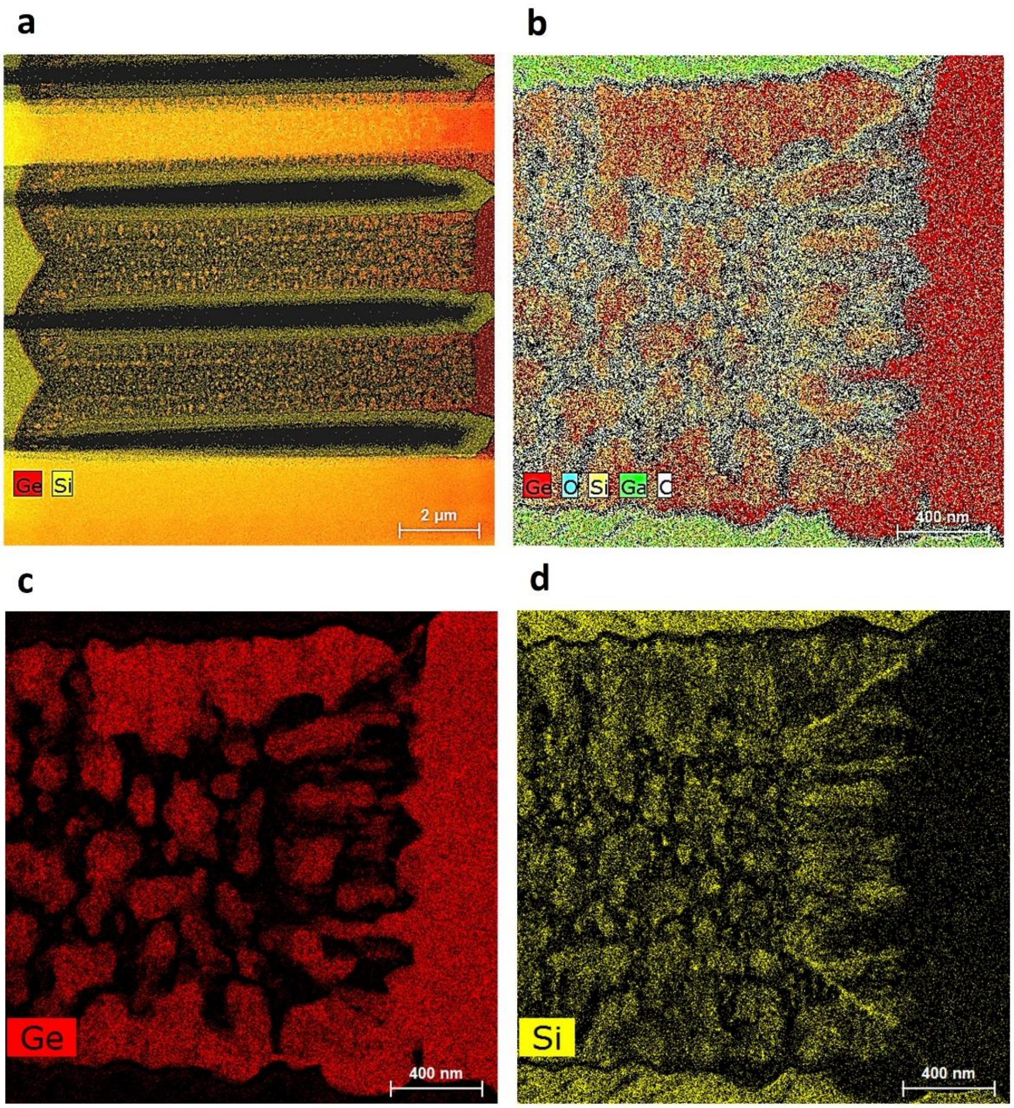

Fig. S6. Energy dispersive X-ray spectroscopy (EDX). EDX mapping of different porous pillars PSiP(70%) on TEM lamella (**a**), complete EDX mapping of the interface Ge/PSiP (**b**), EDX element maps for (**c**) Ge and (**d**) Si elements. As can be observed, the Ge diffused into the porous structure, which would induce a strain on Si. The deformation induced by this infiltration/diffusion of Ge leads to a lattice deformation of the mesoporous Si towards Ge lattice parameter and reduce the strain in this heterostructure.

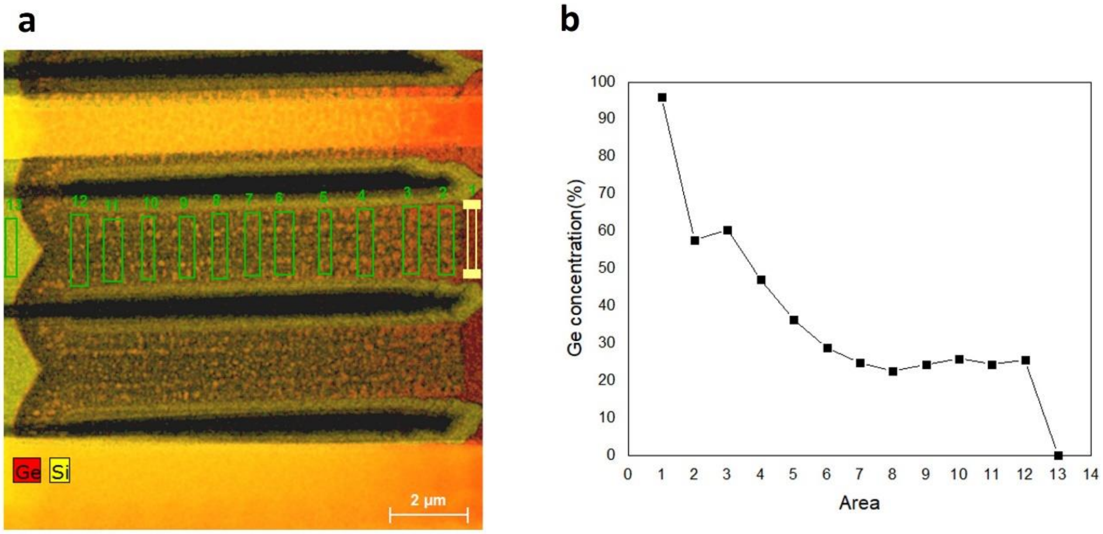

Fig. S7. Ge infiltration in the porous structure. EDX mapping of Ge grow on PSiP(70%) (**a**), Ge concentration over the Si pillar (**b**).

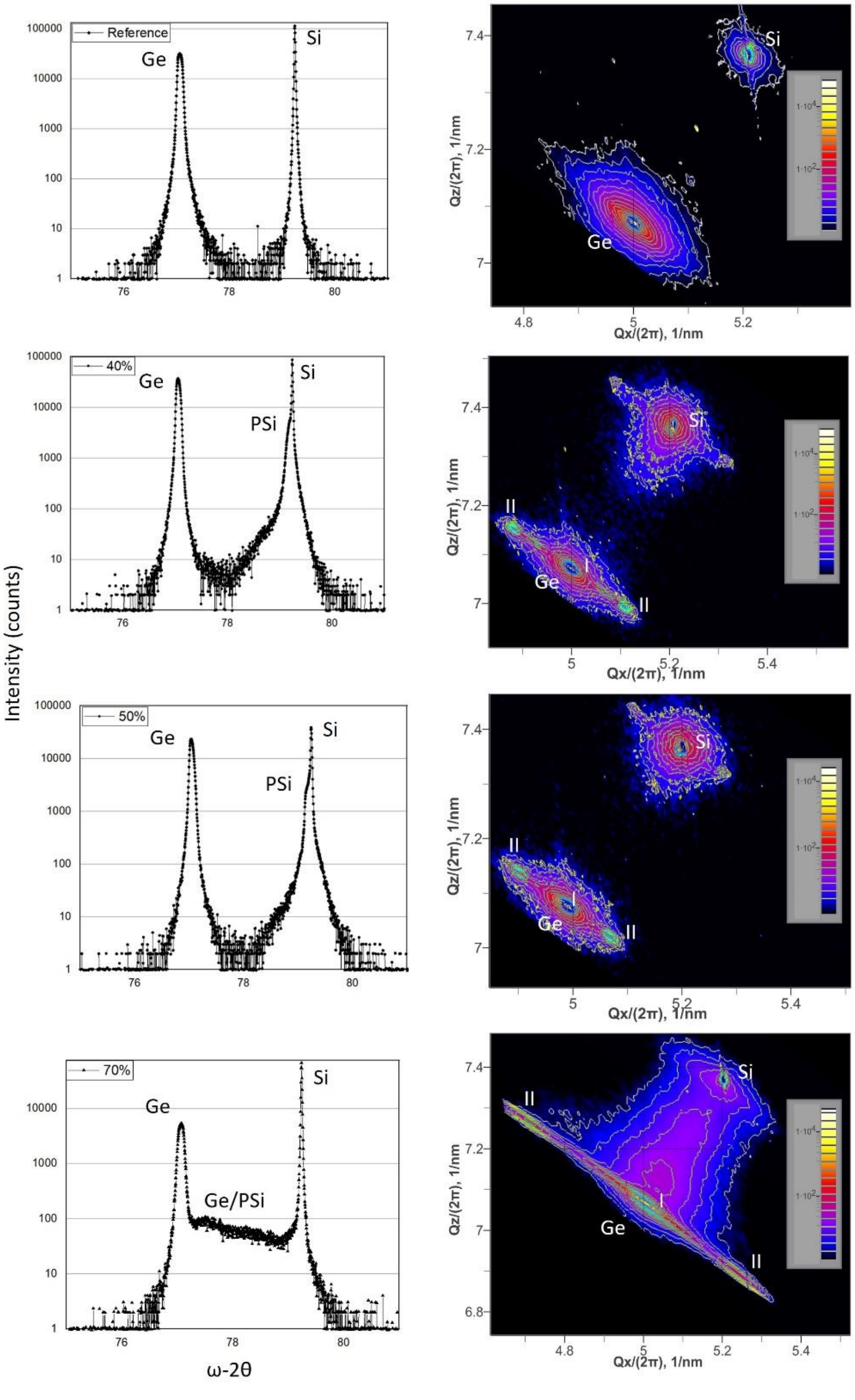

Fig. S8. HRXRD and RSM characterizations of Ge grow on various porous samples. Coupled scans ω-2θ and reciprocal space mapping of the Ge/PSiP heterostructures were performed around the Si(224) reflections.

In order to assess the effect of porosity on TDD, we employed an analytical model allowing to determine the epilayer and dislocation energy evolution depending on the porosity. With such model we can predict, from the theory of compliance[10,51], what would be the ideal porosity required to avoid defect nucleation.
The areal strain energy associated with an isolated screw dislocation is given by[10]:

$$E_D = \frac{Gb^2}{8\pi\sqrt{2}a(x)} \ln\left(\frac{h}{b}\right)$$

We First supposed an epitaxial layer grown on a compliant substrate with lattice mismatch strain $f = (a_s\text{-}a_e)/a_e$, where f is the lattice mismatch strain existing in a coherently strain epilayer, a_s and a_e are respectively the lattice parameter of the substrate and epitaxial layer. In our case we did not consider the curvature at the interface between materials.
Without such curvature, the compliant substrate and the epilayer (microcrystal) are oppositely strained: $\varepsilon_{epi}\text{-}\varepsilon_{sub}=f$
Where ε_{epi} and ε_{sub} represent respectively the in-plane strains in the microcrystal and the substrate. Force balance requires that:
$\sigma_{epi}h_{epi} + \sigma_{sub}h_{sub} = 0$, where h_{epi} and h_{sub} are the thickness of the microcrystal and substrate respectively, and σ_{epi} and σ_{sub} are the in-plane stresses.
The shear modulus of the porous silicon G_p and its Poisson's ratio ν_p can be described by the following empirical rules[38]:

$$G_p = G(1-P)^4$$
$$\nu_p = \nu(1-P)$$

Where G and ν are respectively the shear modulus and Poisson's ration of silicon and P the porosity Considering the elastic constant K, stress can be related to strain as follows
$\sigma_{epi}=K_{epi}\varepsilon_{epi}$
$\sigma_{sub}=K_{sub}\varepsilon_{sub}$
with
$K_{epi} = 2G_{epi}\frac{(1+\nu_{epi})}{(1-\nu_{epi})}$, and $K_{sub} = 2G_p\frac{(1+\nu_p)}{(1-\nu_p)}$
The strain accumulated in the epilayer (Ge microcrystal) will be equal to:

$$\varepsilon_{epi} = f\frac{K_{sub}h_{sub}}{K_{sub}h_{sub} + K_{epi}h_{epi}}$$

And the elastic energy stored per unit area of the interface can be defined as:

$$E_{int} = \frac{K_{epi}h_{epi}K_{sub}h_{sub}}{K_{epi}h_{epi} + K_{sub}h_{sub}}f^2$$

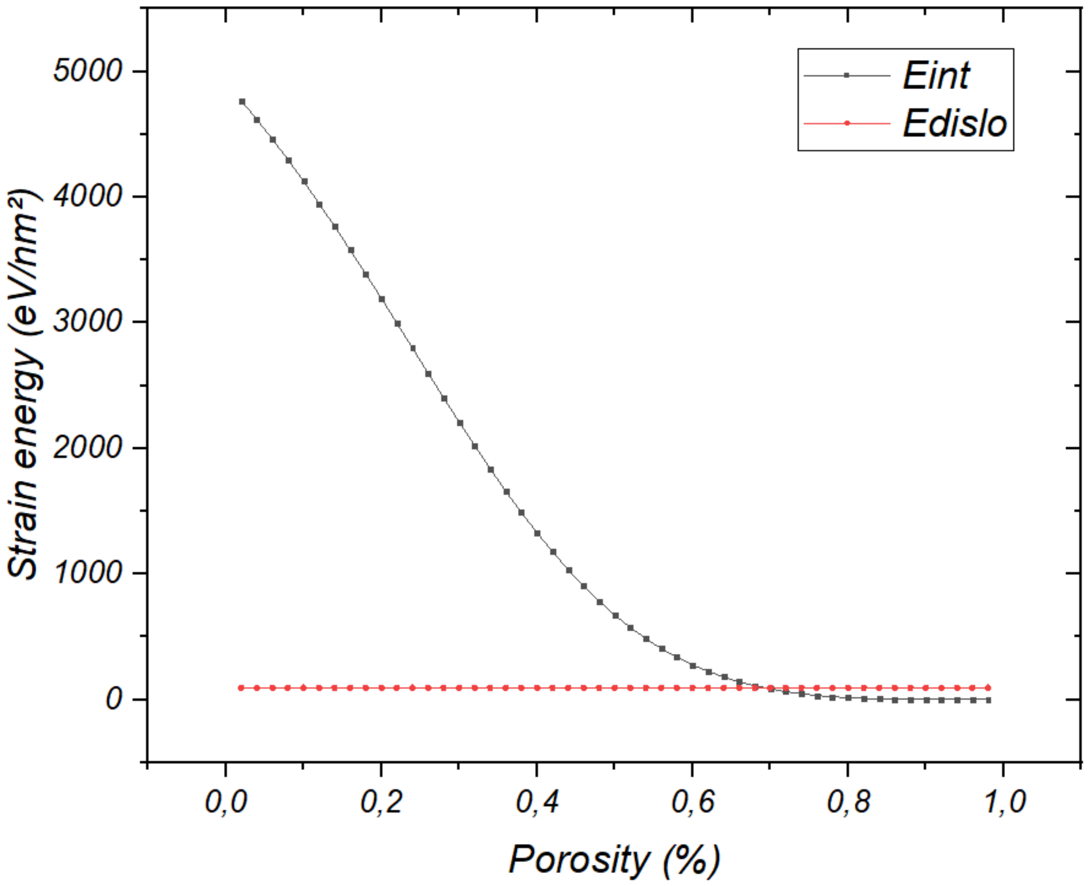

Fig. S9. Analytical model based on theory of compliance. Evolution of the interface and dislocation strain energy depending on the porosity.

As we can observe in Fig. S9, the porosity required to avoid defect nucleation (the strain energy accumulated in the epilayer being inferior to the energy of a dislocation) is around 68%.

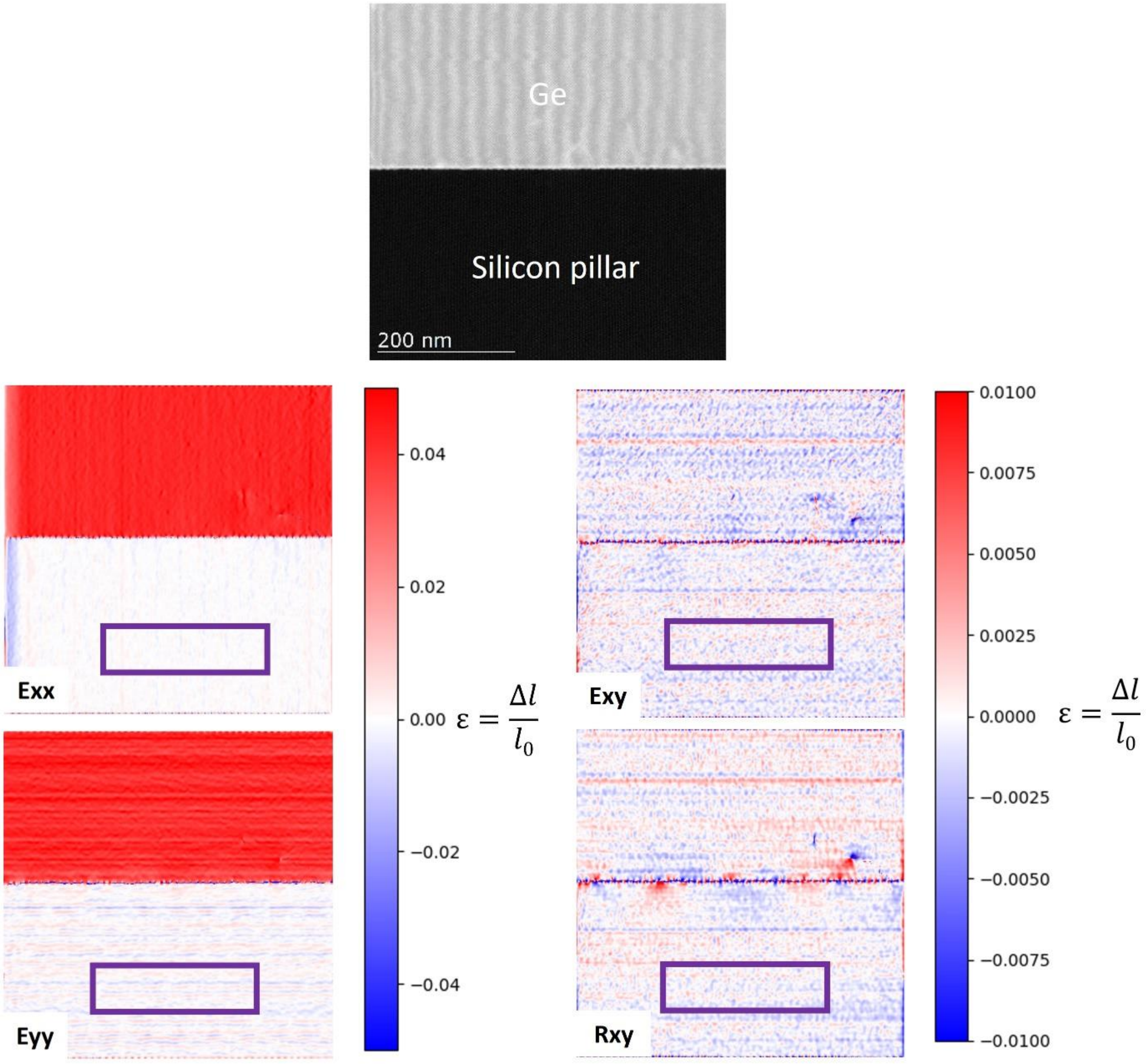

Fig. S10. STEM strain mapping characterization. 2D strain and rotation maps of the Ge/Si pillars reference sample, using purple box region as the arbitrary zero deformation. Horizontal and vertical lines are artefacts from the scanning unit.

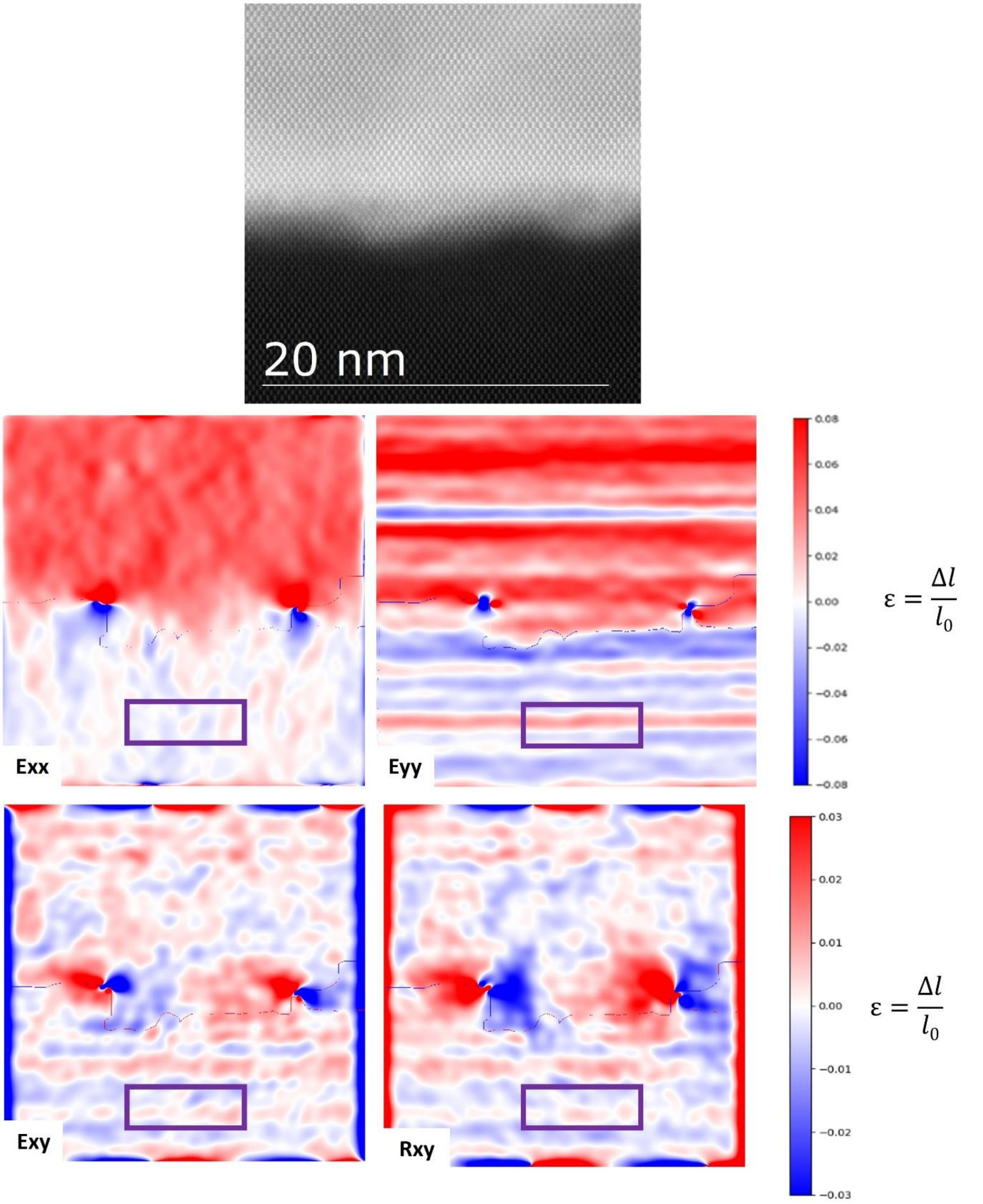

Fig. S11. Misfit dislocations at the interface of Ge/Si pillar. 2D strain and rotation maps of the interface Ge/Si pillars reference sample, using purple box region as the arbitrary zero deformation. Horizontal and vertical lines are artefacts from the scanning unit.

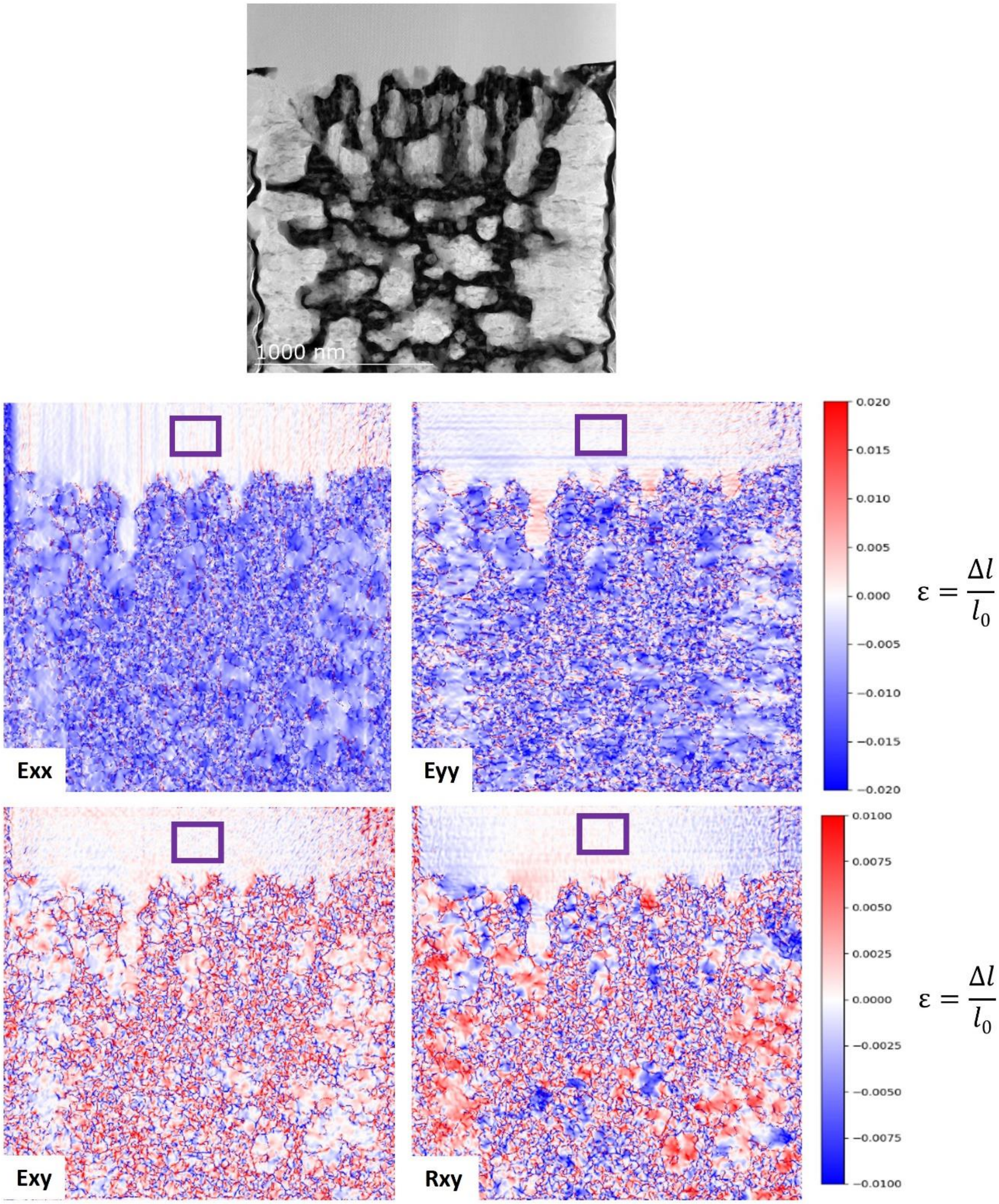

Fig. S12. STEM strain mapping of the porous Si pillar. 2D strain and rotation maps of the porous structure, using germanium region as the arbitrary zero deformation. Horizontal and vertical lines are artefacts from the scanning unit.

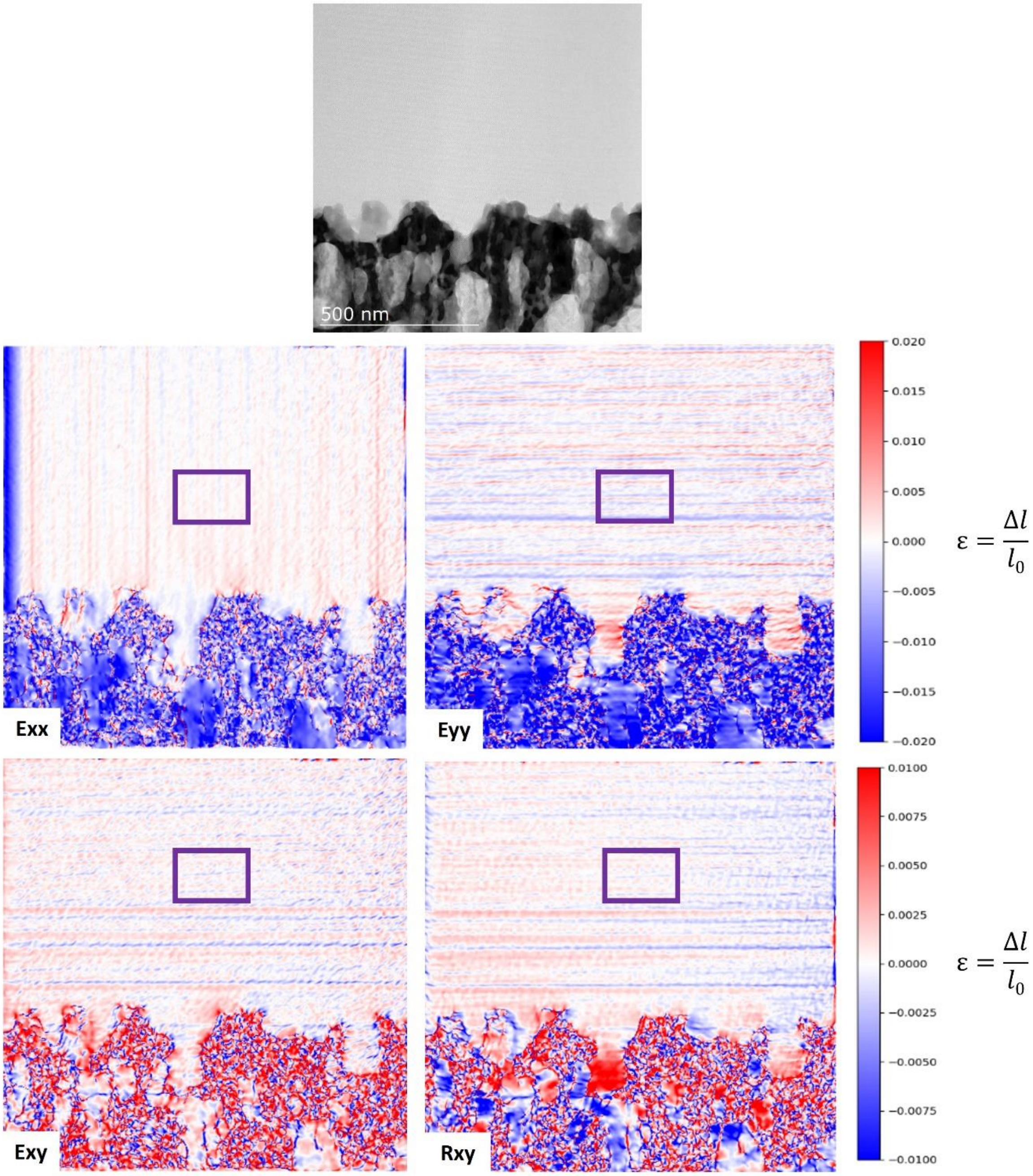

Fig. S13. STEM strain mapping of the interface Ge/PSiP. 2D strain and rotation maps of Ge/PSiP(70%) interface, using germanium region as the arbitrary zero deformation. Horizontal and vertical lines are artefacts from the scanning unit.
